# Interactions of graphene oxide and graphene nanoplatelets with the *in vitro* Caco-2/HT29 model of intestinal barrier

**DOI:** 10.1038/s41598-020-59755-0

**Published:** 2020-02-17

**Authors:** Josefa Domenech, Alba Hernández, Esref Demir, Ricard Marcos, Constanza Cortés

**Affiliations:** 1 Grup de Mutagènesi, Departament de Genètica i de Microbiologia, Facultat de Biociències, Universitat Autònoma de Barcelona, Bellaterra, Spain; 2 Consortium for Biomedical Research in Epidemiology and Public Health (CIBERESP), Carlos III Institute of Health, Madrid, Spain; 3 Antalya Bilim University, College of Engineering, Department of Material Science and Nanotechnology Engineering, 07190-Dosemealti, Antalya, Turkey

**Keywords:** Mechanisms of disease, Genomic instability

## Abstract

Carbon-based nanomaterials are being increasingly used, demanding strong information to support their safety in terms of human health. As ingestion is one of the most important exposure routes in humans, we have determined their potential risk by using an *in vitro* model simulating the human intestinal barrier and evaluated the effects of both graphene oxide (GO) and graphene nanoplatelets (GNPs). A coculture of differentiated Caco-2/HT29 cells presenting inherent intestinal epithelium characteristics (i.e. mucus secretion, brush border, tight junctions, etc.) were treated with GO or GNPs for 24 h. Different endpoints such as viability, membrane integrity, NPs localization, cytokines secretion, and genotoxic damage were evaluated to have a wide view of their potentially harmful effects. No cytotoxic effects were observed in the cells that constitute the barrier model. In the same way, no adverse effects were detected neither in the integrity of the barrier (TEER) nor in its permeability (LY). Nevertheless, a different bio-adhesion and biodistribution behavior was observed for GO and GNPs by confocal microscopy analysis, with a more relevant uptake of GNPs. No oxidative damage induction was detected, either by the DCFH-DA assay or the FPG enzyme in the comet assay. Conversely, both GO and GNPs were able to induce DNA breaks, as observed in the comet assay. Finally, low levels of anti-inflammatory cytokines were detected, suggesting a weak anti-inflammatory response. Our results show the moderate/severe risk posed by GO/GNPs exposures, given the observed genotoxic effects, suggesting that more extensive genotoxic evaluations must be done to properly assess the genotoxic hazard of these nanomaterials.

## Introduction

Carbon-based nanomaterials are being increasingly used, due to their growing relevance in many fields. Their large surface area and their excellent electric conductivity offer many physicochemical advantages that are exploited in many industrial fields, including biomedical applications^1^. Nevertheless, as it occurs with other nanomaterials, its wide use supposes an increased hazard for human health^2^.

One of the most recently discovered carbon-based nanomaterials is graphene. Since its first description in 2004^3^, different modifications have been done in their single-atom-thick sheet of planar sp^2^-bound carbons, creating numerous graphene-based nanomaterials. According to the EU Graphene Flagship project, these materials can be classified according to three different characteristics: (i) the number of graphene layers, (ii) the average lateral size, and (iii) the carbon/oxygen ratio^4,5^. Although a large amount of literature associated with graphene-based nanomaterials has been generated, only a few of them focus on toxicity aspects^2,6^. Hence, this paper focuses on graphene oxide (GO) and graphene nanoplatelets (GNPs) exposure risk, two graphene-based nanomaterials that have shown great potential for biomedical and polymeric composites applications^7,8^.

GO is a highly-oxidized form of graphene, containing different functional groups that easily interact with different biomolecules^9^. This functionalization increases its solubility in water and, in addition, reduces the observed cytotoxicity of graphene and its derivatives. On the other side, GNPs are constituted by short stacks of platelet-shaped graphene sheets that are equivalent to those found in the walls of carbon nanotubes, but with a planar configuration. Notwithstanding the high probability of this contact, only a few *in vivo* studies addressed the hazard of these compounds after oral exposure^10–13^, and only a scarce number of *in vitro* studies have been reported, most of which use the human colon adenocarcinoma Caco-2 cell line as a model of ingestion exposure. Among them, only one used the differentiated Caco-2 cell model^14^, which has proved to be a more reliable model to mimic the small intestine’s enterocyte barrier, both morphologically and functionally^15^. Albeit useful, the simple monolayer of differentiated Caco-2 cells is far away from the complexity of the *in vivo* intestinal barrier, which contains other cell types besides the enterocytes, such as mucus-secreting and immunicity-related cells^16^. Thus, a more complex *in vitro* model of the intestinal barrier should be used to improve our understanding of what occurs in the *in vivo* scenario.

Accordingly, the main aim of this study was to explore the potential effects of GO and GNPs exposures on an *in vitro* model of the intestinal barrier formed by Caco-2/HT29 cell coculture. This model combines the use of enterocyte-like cells (Caco-2) and mucus-secreting cells (HT29) to better mimic the morphology and functionality of the intestinal barrier^17^.

## Methods

### GO and GNPs dispersion and characterization

Both graphene nanomaterials (GO and GNPs, Ref No 763705 and 799092, respectively) were purchased from Sigma-Aldrich (Germany). Both compounds were supplied as water dispersions. Aside from the characteristics provided by the supplier, further characterization was carried out by transmission electron microscopy (TEM), X-ray photoelectron spectroscopy (XPS), Z-sizer, static contact angle measurements, and Profiler 7.0 methodologies.

For XPS, 12 µL of a 100 µg/mL of GO or GNPs water dispersions were dripped onto a gold-coated silica slide and air-dried (n = 2). Measurements were acquired with a SPECS PHOIBOS 150 hemispherical analyzer (SPECS GmbH, Berlin, Germany) at room temperature and 5 × 10^−10^ mbar base pressure, using monochromatic Al Kalpha radiation (1486.74 eV) as the excitation source. Results were analyzed with CasaXPS 2.1.0.1 software.

For TEM analysis, GO and GNPs were previously suspended in 0.05% bovine serum albumin (BSA) diluted in miliQ water and subsequently sonicated at 10% amplitude for 16 min with a S250D Branson Ultrasonics Sonifier Cell Disrupter (VWR, USA) to obtain GNP and GO stock dispersions at 0.95 mg/mL and 1.9 mg/mL concentrations, respectively. TEM grids (n = 2) were immersed in the obtained dispersions and visualized with a JEOL JEM-1400 electron microscope (Jeol LTD, Tokyo, Japan).

Additionally, the hydrodynamic size and Z-potential of previously-sonicated BSA dispersions were also assessed by laser Doppler velocimetry (LDV) and dynamic light scattering (DLS), with a Malvern Zetasizer Nano-ZS zen3600 (Malvern, UK) (n = 3).

Furthermore, we also determined the hydrophobicity of both GO and GNP. We performed static contact angle measurements with an EasyDrop Contact Angle Analyzer (KRÜSS Scientific Instruments, USA). To do this, two different substrates, glass, and methacrylate, were coated with 3–5 layers of dried GO or GNPs by successive drip and evaporation cycles of the water-dispersed materials. Static contact angle measurements were then performed using water droplets. The hydrophobicity of the bare substrates was also measured as a blank, and three different preparations were measured for each condition (n = 3).

For a deeper metrological characterization, graphene nanomaterials stock solutions were diluted at 50 µg/mL in Milli-Q water and air-dried on a silicon substrate. The thickness of GO and GNPs nanoparticles was measured in a mechanical Profiler 7.0 P15 (KLA Tencor, California, USA) device with a stylus radius of 2 µm and an applied force of 2 mg. The step height between the substrate and the nanoparticles was measured for 20 (n = 20) randomly selected GO or GNPs particles scanned at 20 µm/s.

### Endotoxin assay for GO and GNPs

To test for potential endotoxin contamination, in GO and GNPs stock materials, the endotoxin content was measured using the chromogenic *Limulus amebocyte* lysate (LAL) assay [Lonza (QCL-1000), Inc., Walkersville, MD, USA] according to the standard protocol described in the instruction manual, and as previously published by our group^18^. Briefly, all glassware was rendered pyrogen-free by heating overnight at 200 °C. Then, 50 µL of the test sample (or standard) was added to the 96-well plate at 37 °C. At least three wells were used for each sample. The linearity of the standard was verified using lipopolysaccharide (LPS) supplied in the kit. For each assay (n = 3), a standard curve (the concentration ranged from 0.1 to 1 EU/mL endotoxin) was generated over the concentration range 0.116–1.001333 EU/mL and referenced to control standard endotoxin (*Escherichia coli* E50-640). Endotoxin standards and serial dilutions of the sample were assayed in pyrogen-free microtiter plates (Costar No. 3596; Corning, Inc., Corning, NY, USA) in a BioTek Synergy 2 microplate reader (BioTek, Winooski, VT, USA) at 37 °C. Absorbance measurements were taken at 405 nm. Commercially available endotoxin standard (lipopolysaccharide, LPS; 0.5 EU/mL) and LAL water were used as positive and negative controls, respectively. The percentage of the recovery of spiked value was calculated as following (www.lonza.com/qcl1000).

$${\rm{The}}\,{\rm{recovery}}\,{\rm{of}}\,{\rm{spiked}}\,{\rm{value}}\,( \% )=\frac{a-b}{{\rm{c}}}\times 100$$

where ***a*** is the amount of endotoxin found in the spiked sample, ***b*** is the amount of endotoxin found in the sample and ***c*** is the amount of added endotoxin. The recovery of spiked values was calculated as 96.2, 99.2, 100.4, 122.2 and 124.2% for GO (5, 10, 25, 50 and 100 µg/mL) and 101, 101, 101.2, 102.8 and 102.4% for GNPs (5, 10, 25, 50 and 100 µg/mL), respectively.

### Cell culture and reagents

The two different cell lines derived from human colorectal adenocarcinomas used were kindly provided by Dr. Isabella De Angelis (Istituto Superiore di Sanità, Italy) (Caco-2, ATCC HTB-37^™^; or directly purchased from the American Type Culture Collection (ATCC, Manassas VA 20108, USA) (HT29). Cells between passage 35–43 were cultured in Dulbecco’s modified Eagle’s High Glucose medium (DMEM) without sodium pyruvate (Biowest, France) with 1% non-essential amino acids (NEAA) (Biowest, France), 2.5 µg/mL Plasmocin (Invivo Gen, San Diego, CA), and 10% fetal bovine serum (FBS). Cell cultures were kept at 37 °C in a humidified atmosphere of 5% CO_2_ and 95% air and routinely subcultured when reaching 80% confluence, dispersing them with 1% trypsin-EDTA (Biowest, France).

### *In vitro* coculture differentiation

A total of 1.7 × 10^5^ Caco-2 and HT29 cells were seeded on 1.12 cm^2^ PET Transwell membranes with 1 μm pores (Merck KGaA, Darmstadt, Germany) in 12-well plates at a 9:1 ratio and grown for 21 days to induce their differentiation to an enteric barrier model^16^. The monolayer’s integrity was assessed by measuring the transepithelial electrical resistance (TEER) once a week during the period of differentiation. Monolayers were considered suitable for further experiments if their TEER was higher than 300 Ω/cm^2^, according to the ECVAM acceptance criteria^19^.

### Exposure to GO or GNPs

To determine the potential biological effects induced by GO or GNPs, differentiated Caco-2/HT29 barriers were treated with 0, 5, 15, and 50 μg/mL of GO or GNPs for 24 h. Fresh dispersions were prepared by diluting BSA dispersions in DMEM culture medium and 1 mL of either GO or GNPs dispersions was gently poured in the apical compartment of the cocultures. The basolateral chambers’ culture medium was also replaced with fresh DMEM.

### Cell viability determination

The Beckman Coulter method was used to assess cell viability using a ZTM Series Coulter Counter (Beckman Coulter Inc., CA). Briefly, the differentiated monolayers were exposed to GO or GNPs at 0, 5, 10, 25, 50 and 100 μg/mL for 24 h. Subsequently, monolayers were washed with PBS twice, trypsinized for 5 min at 37 °C, and diluted 1:100 in ISOTON (Beckman Coulter Inc., CA). Finally, the detached cells were counted with a Beckman Cell Counter. Two independent experiments (n = 2) were performed.

### Scanning electron microscopy visualization of GO/GNPs on the *in vitro* barrier

The morphological characteristics of GO and GNPs and their interaction with the Caco-2/HT29 monolayer were assessed by scanning electron microscopy (SEM) using the method described by Kucki *et al*.^14^. Briefly, the monolayers were exposed to 50 µg/mL of GO or GNPs, while control samples were cultured only in DMEM. Subsequently, the monolayers were washed with PBS twice and fixed with a modified Karnovsky fixation solution at pH 7.4 (4 g paraformaldehyde, 45 mL PBS without glucose, 50 mL bi-distilled water, and 5 mL glutaraldehyde 50%) at room temperature for 1 h. The samples were then washed with PBS twice and immersed in increasing concentrations of ethanol for 5 min (50%, 75%, 100%, and 100%) for dehydration. Samples were then incubated with 1:1 hexamethyldisilazane (HMDS) and ethanol 100%, followed by treatment with HMDS, dried overnight inside a fume hood and stored until sputter-coated with gold/palladium in an Emitech K550X sputter coater. Samples (n = 2) were visualized with an SEM EVO MA 10 (Zeiss, Germany) instrument.

### Evaluation of the barrier’s integrity

TEER measurements were performed to evaluate the integrity of the barrier using an epithelial Millicell-ERS volt/ohm meter (Merck KGaA, Darmstadt, Germany). To assess the effect of the exposure to GO or GNPs, both chambers of each well were washed with PBS thrice and fresh DMEM was added in the basolateral and apical chambers. Two measurements in different parts of the insert were retrieved from each sample, and TEER values were calculated using the formula TEER = [Ω (cell inserts) – Ω (cell-free inserts)] × 1.12 cm^2^, as previously described^16^. The values for each concentration represent the average of three independent experiments (n = 3). Exposure lasting for 40 min to 20 mg/mL of benzalkonium chloride (Sigma Aldrich, Germany) at 37 °C was used as a positive control.

### Assessment of paracellular permeability

Permeability was measured by assessing the paracellular transport of Lucifer yellow (LY) (ThermoFisher Scientific, USA), as already published^16^. After exposure, the samples were washed thrice with transport buffer Hank’s balanced salt solution (HBSS), (Ca^2+^, Mg^2+^, 10 mM HEPES [4-(2-hydroxyethyl)-1-piperazineethanesulfonic acid], pH 7.4) (Sigma-Aldrich, Germany) and transferred to a new plate with 1.5 mL of HBSS in the basolateral compartment, while 0.5 mL of 0.4 mg/mL LY in HBSS was added to the apical compartment and incubated for 2 h at 37 °C. Finally, the presence of LY was quantified by measuring the fluorescence of 100 μL of the basolateral chamber with a fluorimeter (Victor III, Perkin Elmer, USA) at 405–535 nm as excitation-emission spectrum. A 40 min exposure to 20 mg/mL of benzalkonium chloride (Sigma Aldrich, Germany) at 37 °C and cell-free inserts served as a positive control. Three independent experiments (n = 3) were performed.

### Cellular localization of GO and GNPs by confocal microscopy

After the exposure to GO/GNPs, the monolayers’ nuclei and mucus were stained at room temperature with Hoechst 33342 (ThermoFisher Scientific, USA) at a 1:500 dilution and with Wheat Germ Agglutinin Alexa Fluor 633 (WGA) Conjugate (ThermoFisher Scientific, USA) diluted 1:100, respectively, for 15 min. Afterward, the barriers were washed with DMEM twice, the Transwell’s membrane was cut with a scalpel and placed in Glass Bottom Microwell Dishes (MatTek, USA). GO/GNPs were identified thanks to their light-reflection properties. The images of samples (n = 2) were taken with a Leica TCS SP5 confocal microscope and processed with Imaris 8.2.1 software (Bitplane, AG) to manually mask mucus, nanoparticles, and nuclei in red, green, and blue, respectively.

### Intracellular ROS formation analysis

The induction of oxidative stress was evaluated by detecting intracellular ROS production using the dichloro-dihydro-fluorescein diacetate (DCFH-DA) assay in three independent experiments (n = 3). After the exposure to GO/GNPs, the monolayers were washed with PBS twice and exposed for 1 h at 37 °C to 20 µM DCFH-DA diluted DMEM without serum. A fluorimeter (Victor III, Perkin Elmer, USA) at 490–535 nm excitation-emission spectrum, respectively, was used to quantify the fluorescence emitted by the Caco-2/HT29 barrier cocultures. The positive control was treated with 100 mM H_2_O_2_ for 1 h.

### ROS scavengers mRNA expression analysis

The induction of oxidative stress was also indirectly analyzed by Real-Time RT-PCR to detect variations in the expression of different ROS scavenger genes such as *Superoxide dismutase 2* (*SOD2*), *Heme-oxygenase 1* (*HO1*), and *Glutathione S-transferase P* (*GSTP1*). After the GO/GNPs exposure time, TRI Reagent (Sigma-Aldrich, Germany) was used to extract the total RNA following the manufacturer’s instructions. The samples were subsequently exposed to RNase-free DNase I (Turbo DNA-free kit; Invitrogen, USA) to eliminate residual DNA in the samples. cDNA was obtained by retrotranscription of 100 ng/μL of total RNA using with the High Capacity RNA-to-cDNA kit (Applied Biosystems, USA), and then analyzed by real-time PCR on a LightCycler-480 (Roche, Basel, Switzerland) comparing the expression of the selected genes to an untreated control sample. ß-Actin was used as the housekeeping control gene. We averaged three independent experiments (n = 3). The primers’ sequences are indicated in Table 1.

**Table 1:** Forward and reverse 5′-3′ primer’s sequences of the genes analyzed by Real-Time RT-PCR.

Genes	Forward	Reverse
*Sod2*	5′-GGCCTACGTGAACAACCTGA-3′	5′-GAGCCTTGGACACCAACAGA-3′
*Ho1*	5′-CTCAAACCTCCAAAAGCC-3′	5′-TCAAAAACCACCCCAACCC -3′
*GSTP1*	5′-CCAATACCATCCTGCGTCAC-3′	5′-CAGCAAGTCCAGCAGGTTGT-3′
*β-actin*	5′-GCATGGAGTCCTGTGGCATC-3′	5′-CCACACGGAGTACTTGCGCT-3′

*Note:* Exonic sequences of genes without single nucleotide polymorphisms were chosen to design sense and anti-sense primers for mRNA gene expression analysis. Forward and reverse primers of each gene were designed using the primer designing software Primer Designer version 1.01. All primers were designed with uniform %GC: 55 and Tm of 60 °C for specific amplification.

### Assessment of the genotoxic and oxidative DNA damage

The alkaline comet assay complemented with formamidopyrimidine DNA glycosylase (FPG) enzyme was used to assess the genotoxic and oxidative DNA damage (ODD) levels. In the modified assay, the oxidatively-damaged DNA bases are recognized by FPG, which excises the damaged base/nucleotide, producing a single-strand break. In this way, differences between the levels of DNA breaks in the presence/absence of FPG can help to identify the DNA’s oxidative damage and, consequently, to identify the oxidative potential of a defined compound, including nanomaterials^20^.

Briefly, Caco-2/HT29 barriers were washed with PBS twice, incubated with trypsin-EDTA 1% for 5 min at 37 °C, and centrifuged at 0.3 rcf for 8 min at 4 °C. Cells were resuspended in PBS at 37 °C to obtain 1 × 10^6^ cells/mL and then mixed 1:10 with 0.75% low melting point agarose, dripped on GelBond films (GBF) (Life Sciences, Lithuania) in triplicates, and lysed overnight in lysis buffer (2.5 M NaCl, 0.1 M Na_2_EDTA, 0.01 M Tris Base, 1% Triton X-100, 1% lauroyl sarcosinate, 10% DMSO, and pH 10) at 4 °C. Then, GBF were gently washed twice with enzyme buffer at pH 8 (0.04 M HEPES, 0.1 M KCl, 0.5 mM EDTA, 0.2 mg/mL BSA) for 5 min and 50 min respectively at 4 °C. After that, GBF were incubated with enzyme buffer containing or not the FPG enzyme 1:2500 for 30 min at 37 °C, washed with electrophoresis buffer at pH 13.2 (0.3 M NaOH, 1 mM EDTA) for 5 min, and incubated in electrophoresis buffer for 25 min at 4 °C, to unwind the DNA strands and expose the alkali-labile sites. Subsequently, electrophoresis was carried out at 4 °C at a constant electric tension of 20 V and 300 mA. For the electrophoresis, we used a Fisherbrand Comet Single Cell Gel Electrophoresis Unit (ref. 11817422, Thermo Fisher Scientific, Waltham, MA, USA) with 21.5 × 27.5 × 3.5 cm as active tank dimensions (L x W x H). In addition, the VWR 300 V Programmable Electrophoresis Power Supply (ref. 700–0448, VWR International BVBA, Leuven, Belgium) was also used.

GBF were washed twice for 5 and 10 min respectively with cold PBS, fixed for 1 h in absolute ethanol and air-dried at room temperature overnight. DNA was stained for 20 min at room temperature with 1:1250 SYBR Gold 10 × (Life Technologies, CA, USA) in TE buffer at pH 8 (10 mM Tris Base, 1 mM EDTA). GBF were visualized in an epifluorescent microscope (Olympus BX50) at 20x magnifications. The DNA damage was quantified as the percentage of DNA in the tail with the Komet 5.5 Image analysis system (Kinetic Imaging Ltd, Liverpool, UK). Two independent experiments (n = 2) were performed with duplicates and 100 randomly-selected comet images were analyzed on each sample. As a positive control for genotoxic and ODD damage, monolayers were treated with 200 µM methyl methanesulfonate (MMS) and 5 mM KBrO_3_ for 30 min, respectively.

### Cytokines and chemokines relative levels determination

After the *in vitro* barriers were exposed to GO/GNPs, the cell lysates were collected to determine de levels of 36 different cytokines and chemokines. Protein levels were evaluated in two independent experiments (n = 2) with the Human Cytokine Array kit (ref. ARY005B, R&D Systems, USA). To obtain the cell lysates, the monolayers were washed with PBS twice, solubilized with RIPA 1x lysis buffer (Millipore, Massachusetts, USA) supplemented with complete protease inhibitor cocktail (Roche, Basel, Switzerland), PhosSTOP phosphatase inhibitor cocktail (Roche, Basel, Switzerland), and 1:1000 benzonase nuclease (Merck KGaA, Darmstadt, Germany) and rocked gently for 30 min at 4 °C. Lysates were then centrifuged for 5 min at 14,000 g and the supernatant was tested in the array kit. The assays were carried out following the manufacturer’s instructions. Briefly, the samples were exposed to the Human Cytokine Array Detection Antibody Cocktail for 1 h at room temperature. The array membranes were subsequently blocked for 1 h and incubated overnight with the sample/antibody mixtures at 4 °C. The next day, the array membranes were washed and exposed for 30 min to Streptavidin-HRP at room temperature. Finally, the membranes were washed, soaked with Chemi Reagent Mix and analyzed with GeneGnome (Syngene, Cambridge, UK). The images were analyzed with the Image J software. The barrier was also exposed to 10 µg/mL of lipopolysaccharide (LPS) as a positive control and DMEM as a negative control.

## Results

### GO and GNPs characterization

**Figure 1:**
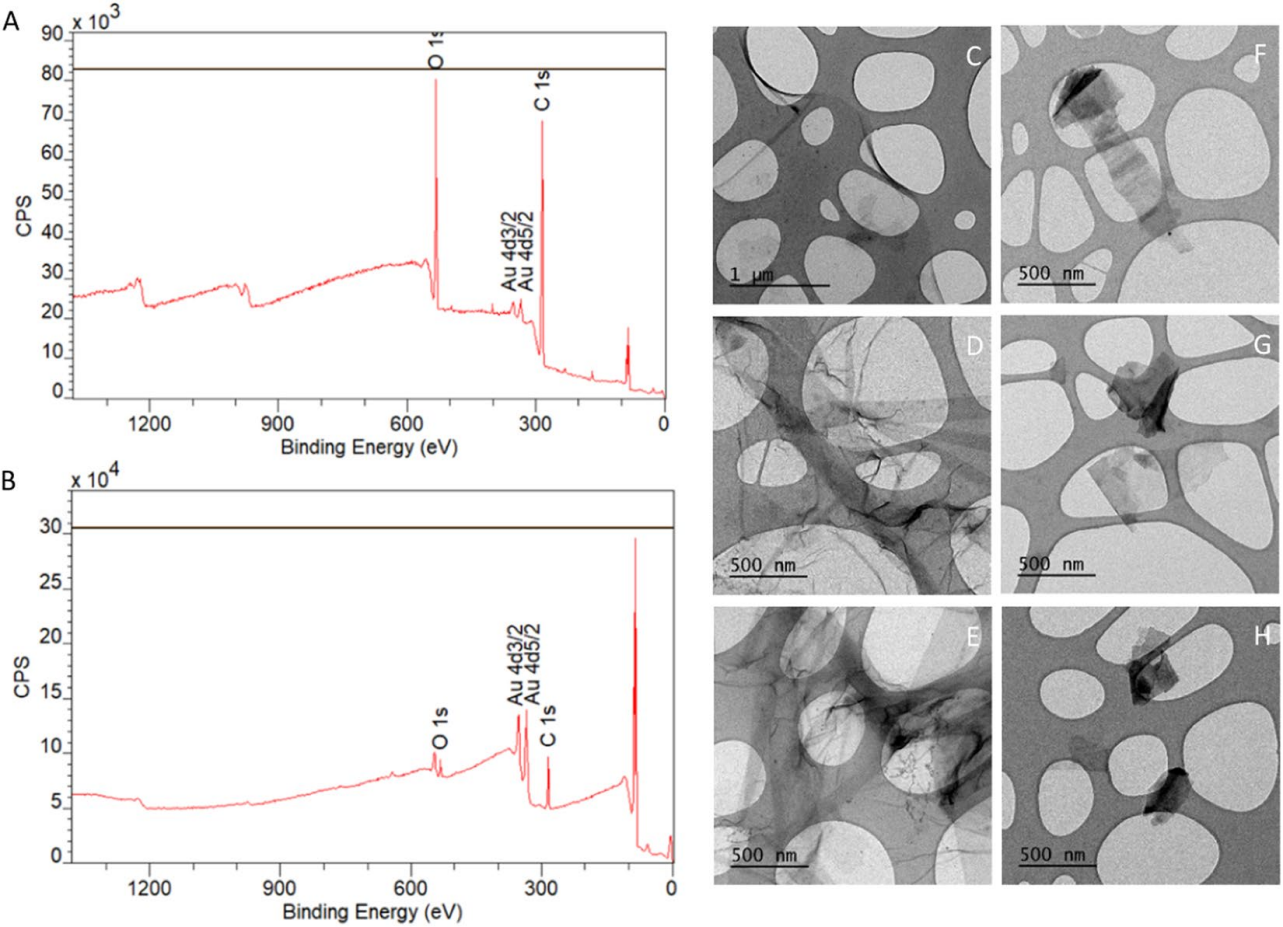
Characterization of GO/GNPs by XPS (n = 2) and TEM (n = 2). Representative diagram of the elemental composition of GO (**A**) and GNPs. (**B**) Carbon and oxygen peaks from GO/GNPs are indicated, gold peaks represent the gold-coated silica substrates. Representative GO (C-E) and GNPs (F-H) TEM images.

XPS was used to characterize the elemental composition of the graphene-derived nanomaterials used, in particular, the carbon-to-oxygen ratio. The obtained results are indicated in Fig. 1A,B. GO dispersions are composed of 75% C and 25% O, while GNPs dispersions consist of 85% C and 15% O. Further analysis were performed by curve fitting to quantify the percentage of C in each of the different bonds with O (Figure not shown). The quantification of the carbon peak for GO dispersion indicates that 40% of the C is linked to the O (58% C-C, 38% C-O, and 4% C=O). On the other hand, the quantification of the carbon peak for GNPs dispersion shows that 13% of the C is linked to the O (87% C-C, 10% C-O, and 3% C=O). As a summary, we get a C/O ratio of 3 and 6 for GO and GNPs, respectively. The percentage that each element was calculated from its peak area, using their relative and sensitive factors (RSF). To calculate the total amount of C and O we took the total area of the corresponding peak. The curve fitting analysis was made to determine the percentage of the element in each of the different bonds.

The overview data shown in Fig. 1 indicates there is no contamination in the samples since only C, O and Au (substrate) were detected in the XPS analysis. The amount of each element cannot be extrapolated from these graphs since the RSF of O are almost three times the RSF of C (RSF O: 2.93/RSF C: 1), that is why the signal of O appears to be very increased in the XPS graph. To have reliable data of the percentage of each element it is necessary to calculate the area under the corresponding peaks and rely on numerical values.

In addition, the morphology of the nanomaterials was characterized by TEM after sonicating the dispersions. As shown in Fig. 1C–E, GO is a single-layered material, while GNPs (Fig. 1F–H) are particles of variable size and irregular shape with a higher electron density, which can be attributed to their multilayer conformation.

Table 2 shows the average hydrodynamic radius and zeta potential of 100 µg/mL GO/GNPs dispersions in 0.05% BSA. The average hydrodynamic radius and the zeta potential were 244.9 ± 7.4 nm and −15.6 ± 0.4 mV for GO and 243.4 ± 1.4 nm and −13 ± 0.5 mV for GNPs, respectively. The zeta potential value indicates that both graphene nanomaterials are prone to aggregation.

**Table 2:** Dynamic light scattering (DLS) and laser Doppler velocimetry (LDV) measurements of GO and GNPs.

Measurement	GO	GNPs
Size (nm) DLS	249.9 ± 7.4	243.4 ± 1.4
PDI (DLS)	0.354 ± 0.052	0.316 ± 0.024
Z-potential (mV) (DLV)	−15.6 ± 0.4	−13.0 ± 0.5
Mobility (µm.cm/V.s) (DLV)	−0.94 ± 0.02	0.79 ± 0.03

Data are represented as mean ± SEM (n = 3).

**Figure 2:**
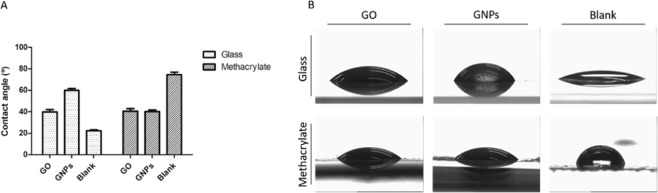
Hydrophobicity measurements by the contact angle. (**A**) Contact angles of water on glass and methacrylate substrates, blank or coated with GO or GNPs. Data are shown as mean ± standard deviation (n = 3). (**B**) Water droplets on glass and methacrylate substrates before and after the coating with GO or GNPs.

The static contact angle measurements showed differences in the level of hydrophobicity between graphene nanomaterials depending on the substrate used (Fig. 2). On the one hand, GNPs appeared to be more hydrophobic than GO when the glass substrate was used, although both graphene materials showed the same static contact angle when the methacrylate was used as a substrate. These results can be explained by the differential behavior that GNPs have on both substrates when forming the cover. This could because the water content of the GNPs is greater than in GO and methacrylate is a highly hydrophobic material. Besides, GO interacts the same way with glass as with methacrylate when forming the cover. This similarity in the behavior of the material on different substrates could explain the fact that we do not see changes in the hydrophobicity of the material depending on the substrate used.

**Figure 3:**
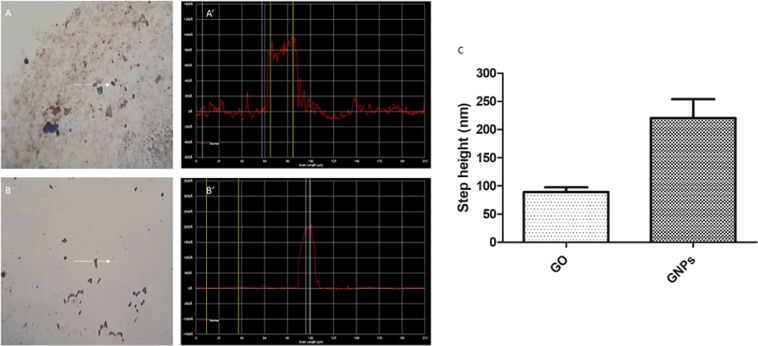
Metrological characterization of graphene nanomaterials by Profiler 7.0. The analysis of a representative GO (**A**,**A’**) and GNPs (**B**,**B’**) particle is shown. White arrows indicate the scanner path over the selected GO or GNPs particle (**A**,**B**). A’ and B’ show the diagram of the measured profile for GO or GNPs representative particle. The mean step height measured for GO and GNPs is indicated in graph C. Data are represented as mean ± SEM (n = 20).

The Profiler 7.0 P15 was used to determine the thickness of graphene materials. A representative measurement obtained from GO and GNPs is shown in Fig. 3A,B, respectively. The analysis of the step height indicated an average thickness of 89.42 ± 8.30 nm and 220.26 ± 33.68 nm for GO and GNPs nanoparticles, respectively (Fig. 3C). These results agree with the fact that GO is a single-layer nanomaterial while GNPs consist of a few-layered material, and they correlate with that observed in TEM images. Twenty randomly selected GO or GNPs particles were analyzed and data are represented as mean ± SEM.

### The endotoxin level of GO and GNPs

**Figure 4:**
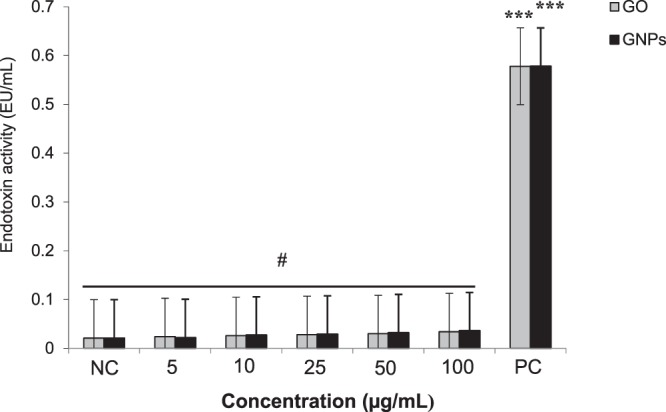
Endotoxin levels (EU/mL) in different concentrations (from 5 to 100 µg/mL) of GO and GNPs detected using the chromogenic *Limulus* amebocyte lysate (LAL) assay. Each point represents a mean of 3 replicates ± standard error (n = 3). The assay was performed according to the kit protocol (Lonza, QCL-1000^TM^). Lipopolysaccharide (LPS) (0.5 EU/mL) was used as a positive control (PC). ^**#**^The endotoxin level of GO and GNPs was below the limit of detection (0.116 EU/mL). ***P < 0.001 when compared to the negative control (NC) (LAL reagent water) using the Student’s *t*-test.

The endotoxin levels of GO and GNPs were evaluated using the LAL assay, and the obtained results are reported in Fig. 4. From these results, we confirmed that the endotoxin levels were below the detectable threshold level of the assay (0.116 EU/mL), which is considered endotoxin-free, in all different concentrations of GO and GNPs. As an example, the observed levels of endotoxins at the highest concentration tested (100 µg/mL) of GO and GNPs were 0.034 and 0.036 EU/mL, respectively. Consequently, we confirmed that our GO and GNPs samples were not contaminated with endotoxins.

### Cytotoxicity assessment

**Figure 5:**
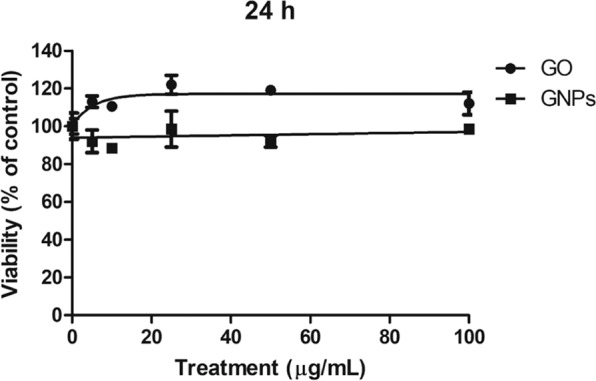
Relative survival of Caco-2/HT29 differentiated cocultured cells after 24 h of exposure to GO/GNPs at concentrations ranging from 0 to 100 µg/mL. The obtained percentage values are the average of the number of cells counted after each treatment, compared to those obtained in the untreated control cultures. Data are represented as the percentage of counted cells compared to the untreated control ± SEM (n = 2).

To evaluate the cytotoxic effects of both GO and GNPs on the Caco-2/HT29 monolayer, a viability assay was carried out after exposing the barriers to 0, 5, 10, 25, 50 and 100 μg/mL for 24 h. As shown in Fig. 5, the barrier presented a high resistance to GO and GNPs cytotoxicity. Cell viability remained constant at all doses, with no significant decrease at the highest tested dose for both GO and GNPs. Considering these results and taking into account that 80 µg/mL is considered a high dose^21^, we selected three concentrations for further experiments: a low concentration of 5 µg/mL, a medium concentration of 15 µg/mL, and a high concentration of 50 µg/mL.

### GO and GNPs interactions with the *in vitro* barrier surface

**Figure 6:**
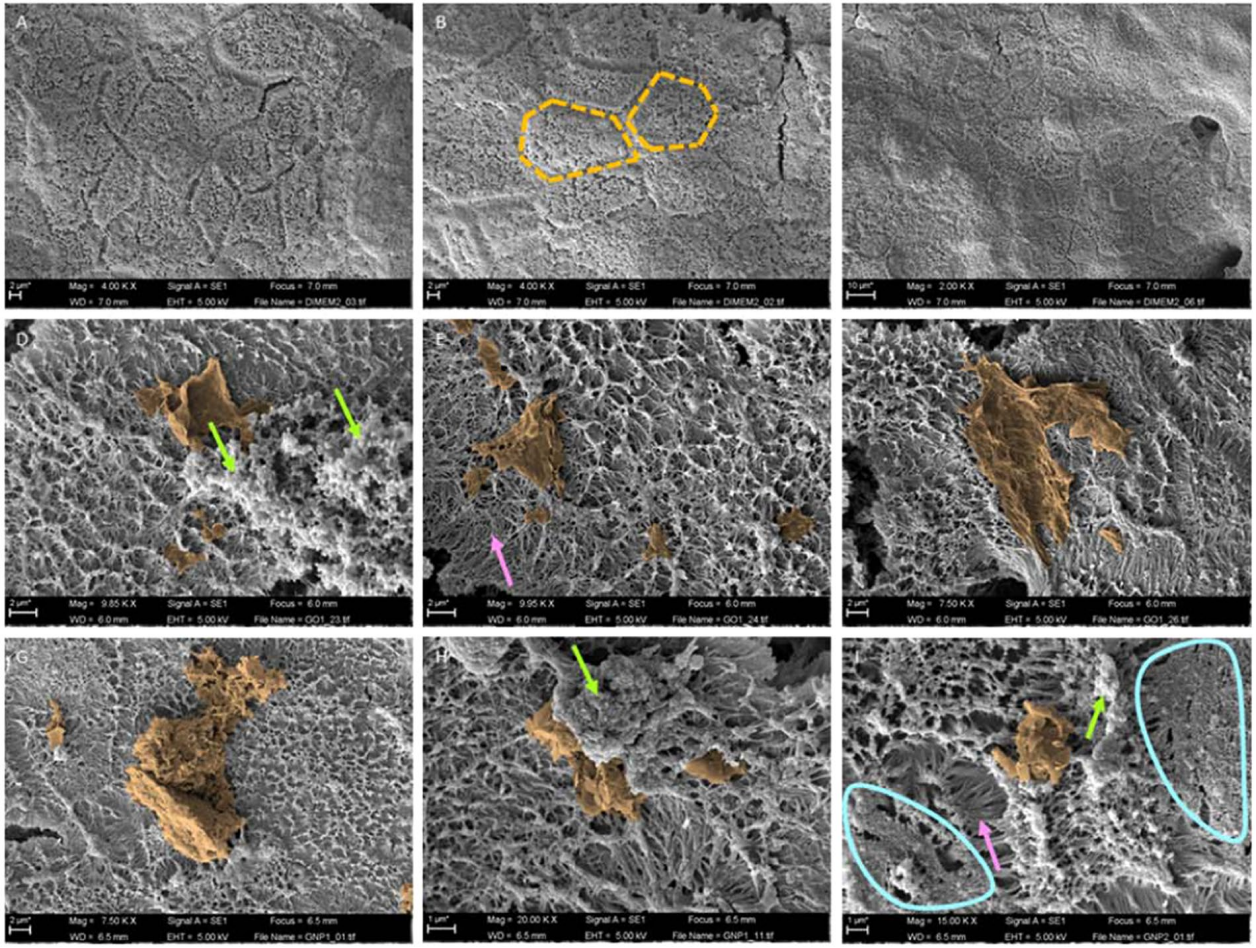
GO/GNPs interactions with the *in vitro* barrier surface. SEM images of differentiated Caco-2/HT29 monolayers without GO/GNPs exposure (**A**–**C**), and after the exposure to 50 µg/mL of GO (**D–F**) or GNPs (**G**–**I**) lasting for 24 h. For better visualization, images were manually modified using Photoshop CC2018 to better delimitate GO/GNPs materials. Orange-dashed lines highlight the hexagonal shape exhibited by the cells, green arrows point out mucus accumulations where the graphene materials are attached. The brush border composed of microvilli (pink arrows) can be seen in the areas surrounded by the blue line. Two different samples for each condition were visualized (n = 2).

To characterize the agglomeration behavior of GO sheets and GNPs, and investigate their interaction with the apical surface of the monolayer, the Caco-2/HT29 cocultures were exposed to the highest tested dose (50 µg/mL) and analyzed by SEM. As indicated in Fig. 6, no changes in the barrier’s morphology were observed when non-exposed monolayers were compared with those exposed to GO or GNPs. In both cases, hexagonal-shaped cells exhibited a dense brush border composed of many microvilli. GO/GNPs appear to be mainly attached to accumulations of mucus and rarely associate to microvilli in the cell surface, indicating a weak interaction between GO/GNPs and the monolayer. In addition, SEM figures confirmed the large, monolayered structure of GO, which is visible over the Caco2/HT29 barrier, maximizing its interaction with the microvilli. On the other hand, most of the GNPs were found as aggregates, although some individual GNPs were also detected.

### GO/GNPs effects on barrier integrity

**Figure 7:**
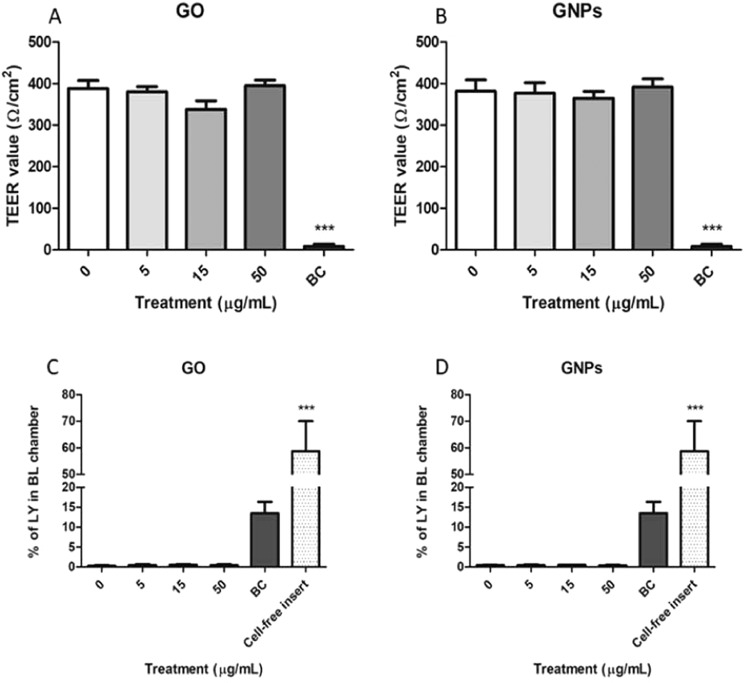
GO/GNPs effects on the Caco-2/HT29 barrier’s stability. TEER values after the exposure to different concentrations of GO (**A**) or GNPs. (**B**) Percentage of LY in the basolateral (BL) chamber after GO (**C**) or GNPs (**D**) exposure. Benzalkonium chloride (BC, 20 mg/mL) was used as a positive control in all experiments. Additionally, a cell-free insert was also used as a positive control in the LY assay. All data are represented as mean ± SEM (n = 3) and analyzed by one-way ANOVA and Dunnett’s multiple comparisons tests (***P < 0.0001).

To evaluate the functional integrity of the barrier, we measured TEER values before and after the exposure to GO/GNPs. As observed in Fig. 7 no significant reduction in TEER values was observed when the barrier was exposed to GO (Fig. 7A) or GNPs (Fig. 7B). Conversely, our positive control, benzalkonium chloride, produced a drastic reduction in the stability of the barrier.

Since a reduction of the integrity of the barrier increases the permeability to a wide range of endogenous and exogenous substances, we also analyzed the change in the flux of LY after GO/GNPs exposure. As observed in Fig. 7, the obtained results reinforce those obtained by measuring the TEER, showing no changes in the stability of the barrier. Thus, no increases in the levels of basolateral LY were observed after the exposure to GO (Fig. 7C) or GNPs (Fig. 7D). As in the previous experiment, our positive control was able to disrupt the integrity of the barrier allowing a significant flux of LY from the apical to the basolateral chamber.

### GO/GNPs uptake and internalization detected by confocal microscopy

**Figure 8:**
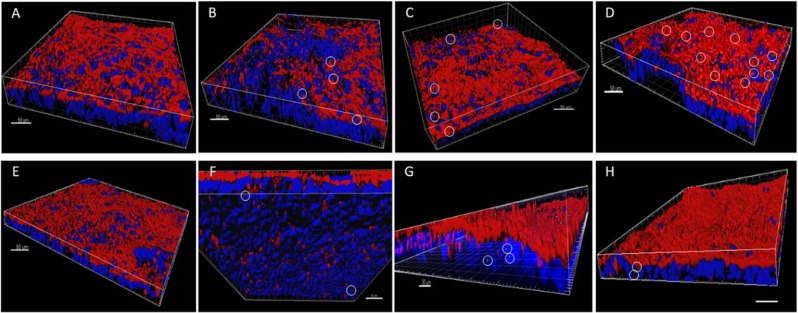
Three-dimensional images acquired with confocal microscopy of the Caco-2/HT29 barrier model after 24 h of exposure to GO (**A**–**D**) or GNPs (E–H). Nuclei (blue) were stained with Hoechst and the mucous shed (red) was stained with WGA. Nanoparticles were manually masked in green. Images (**A**,**E**) correspond to unexposed barriers while (**B**,**F**), (**C**,**G**) and (**D**,**H**) correspond to barriers exposed to 5, 15 and 50 µg/mL of graphene nanomaterials, respectively. Nanoparticles are marked with white circles. Two different samples for each condition were visualized (n = 2).

Confocal microscopy was used to get information regarding the behavior and localization of GO/GNPs in the monolayer, taking advantage of graphene’s reflective properties. As shown in Fig. 8, we were able to detect changes in the nanoparticles’ localization depending on the nanomaterial used. All the detected GO nanoparticles were embedded into the apical mucous layer of the barrier at all the tested concentrations (Fig. 8A–D), and no uptaken GO was detected inside the cells. On the other hand, GNPs were embedded into the mucus and also inside the cells, independently of the concentration assayed and not in a concentration-dependent manner (Fig. 8E–H). This would confirm that GNPs were able to cross the mucus barrier and enter the cells. Taking into account the differences between GO and GNPs regarding size and number of layers, it seems that the ability of internalization is modulated by the nanoparticle size.

### Intracellular oxidative stress induction

**Figure 9:**
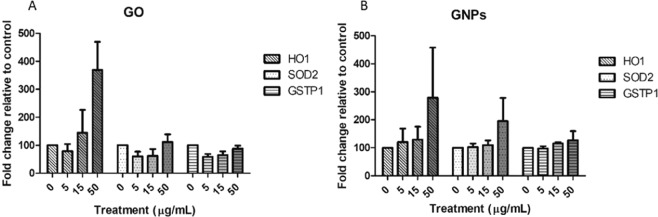
Relative mRNA expression analysis using Real-Time RT-PCR of *HO1*, *SOD2*, and *GSTP1* genes. The Caco-2/HT29 coculture barrier model was treated with 0–50 μg/mL of GO (**A**) and GNPs (**B**) for 24 h. Data are represented as mean ± SEM (n = 3) and analyzed by the one-way ANOVA with a Tukey’s post-test.

**Figure 10:**
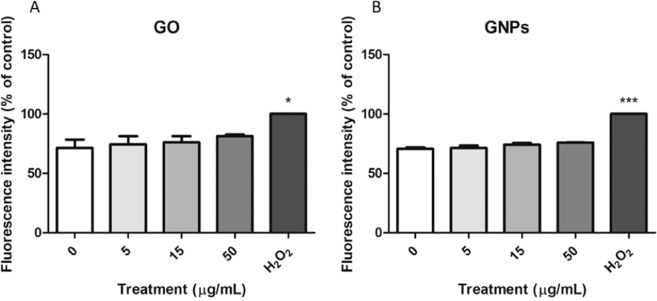
Relative analysis of the intracellular ROS production in the Caco-2/HT29 cocultures treated with 0–50 μg/mL of GO (**A**) and GNPs (**B**) for 24 h, using the DCFH-DA assay. 100 mM H_2_O_2_ was used as a positive control. Data are represented as mean ± SEM (n = 3) and analyzed by one-way ANOVA with a Tukey’s post-test (***P < 0.0001, *P 0.0126).

Changes in the expression of three ROS-related genes were assessed by Real-Time RT-PCR. As shown in Fig. 9, we could not detect significant changes in the expression of the selected genes for any of the assayed exposures. However, a slight concentration-dependent tendency to increase the expression of the ROS-related genes was observed for both nanomaterials. To confirm these results, intracellular ROS levels were measured by using the DCFH-DA assay, and the results are indicated in Fig. 10. As observed, we could not detect any increase of intracellular ROS in the Caco-2/HT29 barrier cells after the exposures to GO/GNPs.

### DNA damage as detected with the comet assay

**Figure 11:**
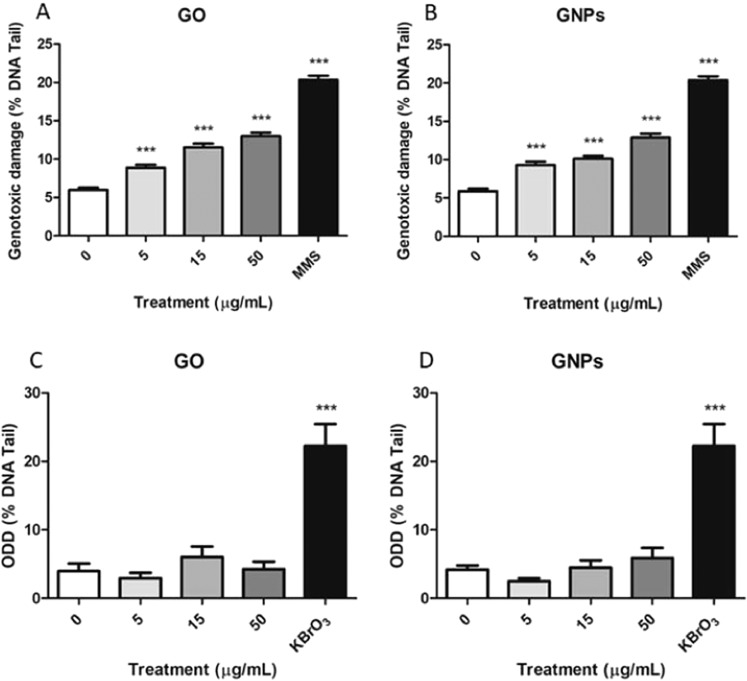
Genotoxic damage observed by the comet assay after 24 h of GO (A) or GNPs (B) exposure, 200 µM MMS was used as a positive control. Oxidative DNA damage (ODD) detected using the FPG enzyme, observed after 24 h of GO (C) or GNPs (D) exposure, 5 mM KBrO_3_ was used as a positive control. Data are represented as mean ± SEM (n = 2) and analyzed by the one-way ANOVA with a Dunnett post-test (***P < 0.0001).

As shown in Fig. 11A,B, the percentages of DNA found in the tail of the cells exposed to GO and GNPs were significantly higher than those observed in untreated cells. In addition, the genotoxic damage induced by both treatments was dose-dependent. As observed, the positive control used, 200 µM MMS, gets a clear increase in the levels of DNA breaks. On the other hand, when the FPG enzyme was used to detect oxidized DNA bases and transform them into single-strand breaks after their excision, no significant differences relative to the control samples were observed. This would indicate that the DNA breaks observed after the exposures to GO or GNPs do not derive from oxidatively-damaged DNA bases. As observed in Fig. 11C,D, KBrO_3_ produced significant increases of the ODD, demonstrating the suitability of the assay to detect oxidatively-damaged DNA.

### Relative levels of cytokines and chemokines secreted after CO/GNPs exposure

**Figure 12:**
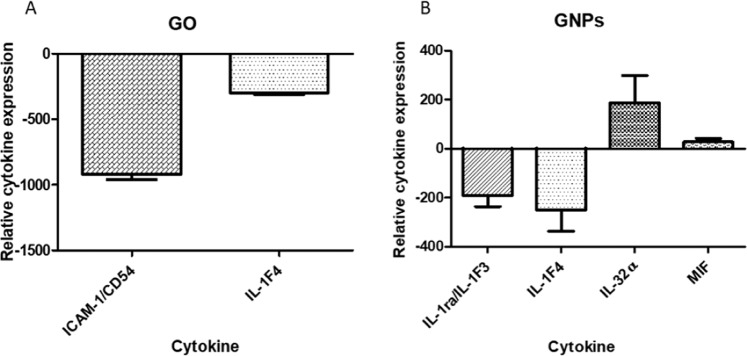
Relative cytokine expression of the Caco-2/HT29 barrier after the exposure to 50 µg/mL of GO (**A**) or GNPs (**B**) for 24 h. Data represented is the result of subtracting the negative control to each value. Data are represented as mean ± SEM (n = 2) and analyzed by Student’s t-Test.

The relative levels of 36 cytokines and chemokines were determined after the exposure of the Caco-2/HT29 monolayer to 50 µg/mL of GO/GNPs by using a Human Cytokine Array kit. After the exposure to GO, a poor response was observed, since relevant changes were detected in only two cytokines (ICAM-1/CD54 and IL-1F4), as shown in Fig. 12A. On the other hand, changes in four cytokines were detected after the exposure of the barrier to the highest concentration of GNPs (Fig. 12B). As observed, the levels of expression of IL-1ra/IL-1F3 and IL-1F4 showed a decrease in a similar way to that observed in the case of GO. In addition, increases in the expression of IL-32α, and to a lower extent in MIF, were also detected after the exposure to GNPs.

## Discussion

Ingestion represents an important entry exposure route for nanomaterials. This is obviously true for those nanomaterials used by the food industry such as additives, antimicrobials or food packaging. However, it should be pointed out that airborne nanomaterials can also be ingested by oral breathing if they are deposited on foods, or during the clearance process of inhaled nanomaterials. Accordingly, good *in vivo/in vitro* models are required to evaluate the potential risks associated with this type of exposure route^22^, as well as considering the potential biotransformation that nanomaterials could suffer in response to degradation by digestive fluids^22,23^. The potential effects of *in vitro* digestion on the physicochemical characteristics of graphene compounds (few-layer graphene and graphene oxide) have been previously evaluated. No structural changes/degradation of these nanomaterials were detected, suggesting that they are biopersistent when administered by the oral route^23^.

As a well-established *in vitro* target of ingestion exposures, the Caco-2 cell line represents the most frequently used cell model of the intestinal tract^24^. Although most of the studies frequently use Caco-2 cells in their undifferentiated state as a model system, these cells have the ability to undergo a differentiation process leading to structured cell monolayers, morphologically and functionally equivalent to the enterocytes of the small intestine^24^. Nevertheless, since the monolayer of Caco-2 cells does not completely mimic the complexity of the *in vivo* intestine, the use of complementary cell lines has been proposed to better imitate the structure/function of the human intestine. Thus, the coculture with HT29, cells characterized by their ability to produce and secrete mucus, and/or the use of human Raji-B lymphocytes, as cells that induce Caco-2 to differentiate into M-like cells, have been proposed to improve the resemblance of the model^16^.

In humans, exposure to graphene-based nanomaterials seems to take place mainly at work sites, and it occurs via inhalation, ingestion, cutaneous or ocular exposure. Few *in vivo* studies have been conducted to determine the risk of such types of exposures, so those using ingestion are scarce^2^. In the mouse model, when mothers and offspring were exposed to GO via drinking water, developmental changes were observed in the offspring. Interestingly, changes in the morphology of the microvilli of the intestinal cells were also observed, although these changes normalized with the cessation of the exposure^11^. In a preliminary study, rats exposed to GO via drinking water presented significant nephrotoxicity associated with increased activities of superoxide dismutase, catalase, and glutathione peroxidase in their kidneys. Nevertheless, that study did not analyze the effects induced by GO at the intestinal barrier level^12^. Finally, a biodistribution study of GO in the body of mice after oral administration was carried out by labeling GO with ^125^I. Radioactivity was detected in many organs, but mainly in the liver. All these data would confirm that, after *in vivo* exposures, GO can reach the main organs through intestinal absorption^11^.

Regarding *in vitro* approaches using Caco-2 cells, six studies have been conducted in undifferentiated cells testing graphene-based compounds^14,23,25–28^, and only two used differentiated Caco-2 cells monolayers^14,23^.

With undifferentiated cells, DNA strand breaks were detected by the comet assay related to the nanomaterial size, although no cytotoxicity was observed after GO exposures lasting for 24 h. For micrometer-sized GO, the genotoxicity was positively correlated with the concentration, while for nanometer-sized GO, the higher degree of genotoxicity was detected at the lowest concentration assayed^25^. The low toxicity of GO after exposures lasting for 24 h was also observed by Nguyen *et al*.^26^. Nevertheless, a comparative toxicity study using five different carbon-based nanomaterials showed that GO was the most toxic (and also the smallest in size), but in exposures lasting for 5 days. In addition, no changes in the oxidative stress status were observed after 24 h of GO exposure. Interestingly, the use of TEM permitted to visualize their uptake by Caco-2 cells^27^. The low toxicity was also reported for four different GO and GNPs species, showing that changes in their physicochemical properties do not modulate the cytotoxicity. In that study, contrarily to the results observed in one of the previous studies, the authors found that GO and GNPs exposures were able to induce a significant increase in the levels of intracellular ROS^21^. The last and more recent study deals with the uptake of MWCNTs and GO by endocytosis. After knocking down three members of Rho GTPases, in such conditions the endocytosis of GO was increased in Caco-2 cells, showing that GO uptake was modulated by caveolins^28^.

One of the studies that used monolayers of differentiated Caco-2 cells mainly focuses on the uptake of GO. Results showed that the uptake mainly depends on the cell differentiation status, with undifferentiated cells showing higher internalization^14^. In fact, the different behavior of differentiated *vs* undifferentiated cells has been reported for other nanomaterials like CeO_2_NPs^29^. These differences in the internalization of nanomaterials could be due to the small intestine-like structure shown by differentiated cells. Thus, a more complex cell structure and morphology with microvilli in the apical cell structure could complicate the intake of NPs. Kucki *et al*.^14^ pointed out that barrier models including different cell types are needed to improve the model.

Given the previous information found in the literature, in this study we worked with a complex Caco-2/HT29 barrier model, trying to better mimic the interaction between graphene nanomaterials and the gastrointestinal tract. This model has recently shown to be useful to understand the role of the different shapes of TiO_2_NPs in their uptake and their consequent biological effects^17^. In addition, this model demonstrated that the cellular uptake of AgNPs showed a time-dependent and concentration-dependent increase^30^. Using this model, we have confirmed the low cytotoxicity of both GO and GNPs on the barrier’s cells. Additionally, both of these graphene-based nanomaterials were unable to affect the functional/structural integrity of the barrier, since TEER values and LY flux were not affected by GO/GNPs exposures. It should be emphasized that no previous studies have analyzed the integrity of the intestinal barrier after GO/GNPs exposure. Interestingly, a previous study using MWCNT showed that exposures to low concentrations of this carbon nanomaterial improved the quality of the barrier affected by *Escherichia coli*, by reducing the tight junction permeability and restituting the integrity of the microvilli structure in the apical cell surface^31^. The role of pristine and oxidized MWCNT interactions on Caco-2 monolayers was also evaluated by other authors^32^, showing the absence of uptake, although some damage in microvilli was observed.

The demonstration of the uptake of nanoparticles by undifferentiated Caco-2 cells using TEM methodologies can suppose a potential underestimation due to the granulated nature of the Caco-2’s cytoplasm, which can mask some internalized nanoparticles^33^. To avoid potential misinterpretations, the use of confocal microscopy can be very helpful, especially when differential staining is used to distinguish if NMs are located on the surface of the epithelia or if they are internalized into the cells’ cytoplasm or their nuclei^34,35^. Using this approach, we have been able to demonstrate that GNPs were internalized, while most of the GO remained attached to the mucous shield. Our results reinforce the usefulness of confocal microscopy to analyze the fate of nanomaterials inside the cells. Since size and the number of layers are the differential characteristics between GO and GNPs, both characteristics could be involved in the different observed uptakes. Another important parameter to consider when determining uptake parameters is adhesion. Different studies have pointed out that the adhesive force among biological membranes and graphene decreases at higher oxidation states^36,37^. Furthermore, hydrophobic materials are more likely to adhere to the lipid bilayers of cell membranes^38,39^. These data are consistent with our observations by XPS analysis, which show that GNPs would be more prone to adhere to the Caco-2/HT29 membranes, while GO would have lower adhesion to cells. All the different parameters evaluated, including the measured oxygen-carbon ratio, thickness, and hydrophobicity, support the higher cell adherence of GNPs and, consequently, its higher uptake.

Among the different biological effects usually associated with nanomaterials exposure, their ability to induce intracellular oxidative stress should be highlighted. Imbalances in the redox state of the cells are associated with different human pathologies, such as respiratory diseases, allergies, cardiovascular disease, and growth disorders^40^. The induction of intracellular ROS is a biomarker that must be included when the potential health effects of a specific nanomaterial are evaluated. In our case, neither GO nor GNPs were able to induce changes in the intracellular levels of ROS. Although our results contradict what it was found by Kucki *et al*.^21^, they would support the results reported by Saha *et al*.^27^, which tested five different types of carbon materials which varied in size, shape, surface area, surface functionality, and conductivity. At this point, it should be reminded that both studies were carried out using undifferentiated Caco-2 cells, which show important differences in uptake with differentiated Caco-2 cells. No other studies evaluating intracellular ROS levels in differentiated Caco-2 cells after GO/GNPs exposure have been reported until now, highlighting the relevance of our findings. In addition, no studies assessing the levels of intracellular ROS in differentiated Caco-2 cells have been reported for other carbon-based nanomaterials.

In our study, no DNA oxidative damage was detected, confirming the results showing the absence of significant increases in the levels of intracellular ROS. Nevertheless, we were able to observe the induction of DNA damage as measured by the comet assay. This type of damage mainly reflects DNA breaks induced by a ROS-independent mechanism. Although we can assume that some DNA-GNPs direct interaction occurs, given the significant cellular uptake observed, results with GO are more speculative. Regarding potential mechanisms involved in the ability of GO to induced genetic damage, a previous study has identified the down-regulation of key genes involved in DNA damage response and repair mechanisms using a cDNA microarray and confirmed this genotoxic effect using the comet assay^41^. In a similar way, the interaction between GO and p53 has also been proposed as another mechanism involved in the potential genotoxic role of GO^42^. Nevertheless, in both cases, it is assumed that physical contact must exist between DNA and GO, but it is not clear that this occurs in our experimental conditions. It is possible that small pieces of GO enter the cell and escape the detection by confocal microscopy, or that the analyzed time frame does not allow the detection of ROS production. Alternatively, other mechanisms besides oxidative stress and physical interaction could act to produce the observed DNA damage.

A couple of studies have addressed the pro- and anti-inflammatory effects of graphene-related nanomaterials, taking into account their potential use as novel biomaterials^43,44^. Cytokines, as small nonstructural proteins involved in cell trafficking during inflammation, are indicative of an immune response. The behavior of the cytokines is complex since they have both pro-inflammatory and anti-inflammatory potential. In our study, we have found a decrease in the expression of the major pro-inflammatory IL-1 cytokines after the exposure to both GO and GNPs. This would agree with the observations made on macrophages exposed to GO, where a dose-dependent inhibition of interleukin IL-1 and IL-6 were observed^45^. This would also agree with the results obtained using a nanocomposite formed by GO and AgNPs, where the primary murine macrophages exposed did not produce inflammatory cytokines^46^. In our case, we have also found a lowered expression of ICAM-1 in the cells exposed to GO. The expression of this cytokine matches well with the behavior of IL-1 and, although no studies have been reported covering the effects after exposures to carbon-based nanomaterials, lowered expression of this cytokine was observed in rheumatoid arthritis patients after the exposures to anti-rheumatic drugs^47^. A similar effect has been documented in mice, where the induction of acute renal injury was protected by the bioactive flavonoid Luteolin, showing anti-inflammatory and renal protection effects^48^.

Interestingly, a different response was observed after the exposure to GNPs, where an enhanced expression of IL-32 was observed. The role of IL-32 in inflammation is not clear. Although IL-32 is upregulated in individuals with numerous inflammatory diseases, it has been pointed out that it can be downregulated in other inflammatory diseases including HIV infection disease, asthma, metabolic disorders, neuronal diseases, and experimental colitis^49^. Among the different roles assigned to IL-32, its ability to regulate other interleukins such as TNF-α and IL-1 must be highlighted. In this way, it has been observed that IL-32 negatively regulates the production of IL-1^50^. This would match with our observed results after the exposure to GNPs. Taken together, our overall results after GO/GNP exposure do not show an inflammatory response in the Caco-2/HT29 coculture, which would support their potential use as promising biomaterials for bone tissue engineering, by inducing a beneficial immunomodulatory bone environment^51^.

## Conclusion

After using a wide set of approaches to evaluate the hazard posed by GO and GNPs exposure, our conclusion is that their associated risk can be considered moderate/severe. No cytotoxic effects in the Caco-2/HT29 cells were observed, and no damaging effects were detected in the integrity of the barrier or in its permeability. Additionally, no oxidative damage induction was detected using different approaches: gene expression, DCFH-DA assay, and detection of oxidized DNA bases. Furthermore, minimum changes in the levels of the expression of cytokines were observed, showing a mild anti-inflammatory response. Nevertheless, cellular uptake, mainly of GNPs, was observed in our model of the intestinal barrier, and levels of DNA damage were statistically-significant when the coculture was exposed to both GO and GNPs. According to the relevance of the genotoxic damage results, further genotoxic evaluations must be done, mainly using other endpoints detecting fixed DNA damage in addition to the damage observed in the comet assay that, as primary DNA damage, can be easily detected and repaired.

## Data Availability

The data supporting our conclusions are included in the main body of the manuscript.
